# Detoxification, Hydrogen Sulphide Metabolism and Wound Healing Are the Main Functions That Differentiate Caecum Protein Expression from Ileum of Week-Old Chicken

**DOI:** 10.3390/ani11113155

**Published:** 2021-11-04

**Authors:** Jiri Volf, Jana Rajova, Vladimir Babak, Zuzana Seidlerova, Ivan Rychlik

**Affiliations:** Department of Microbiology and Antimicrobial Resistance, Veterinary Research Institute, Hudcova 296/70, 621 00 Brno, Czech Republic; rajova@vri.cz (J.R.); babak@vri.cz (V.B.); seidlerova@vri.cz (Z.S.); rychlik@vri.cz (I.R.)

**Keywords:** hydrogen sulphide, wound healing, caecum, ileum, stress response, chicken

## Abstract

**Simple Summary:**

Although the ileum and caecum represent adjacent parts of the gastrointestinal tract, both compartments differ by function as well as inner environment parameters such as oxygen availability or density of colonising microbiota. As the function of a particular tissue is generally reflected by protein expression, mass spectrometry proteomics was used to characterise expressed proteins of both segments of the gastrointestinal tract. Differentially expressed proteins were identified and grouped according to biological processes specific to both gut compartments.

**Abstract:**

Sections of chicken gut differ in many aspects, e.g., the passage of digesta (continuous vs. discontinuous), the concentration of oxygen, and the density of colonising microbiota. Using an unbiased LC-MS/MS protocol, we compared protein expression in 18 ileal and 57 caecal tissue samples that originated from 7-day old ISA brown chickens. We found that proteins specific to the ileum were either structural (e.g., 3 actin isoforms, villin, or myosin 1A), or those required for nutrient digestion (e.g., sucrose isomaltase, maltase–glucoamylase, peptidase D) and absorption (e.g., fatty acid-binding protein 2 and 6 or bile acid–CoA:amino acid N-acyltransferase). On the other hand, proteins characteristic of the caecum were involved in sensing and limiting the consequences of oxidative stress (e.g., thioredoxin, peroxiredoxin 6), cell adhesion, and motility associated with wound healing (e.g., fibronectin 1, desmoyokin). These mechanisms are coupled with the activation of mechanisms suppressing the inflammatory response (galectin 1). Rather prominent were also expressions of proteins linked to hydrogen sulphide metabolism in caecum represented by cystathionin beta synthase, selenium-binding protein 1, mercaptopyruvate sulphurtransferase, and thiosulphate sulphurtransferase. Higher mRNA expression of nuclear factor, erythroid 2-like 2, the main oxidative stress transcriptional factor in caecum, further supported our observations.

## 1. Introduction

The chicken intestine consists of the small intestine, colon, and paired caecum. The colon in chickens is quite short, and major digestion and nutrient absorption occur in the small intestine. The function of the caecum in chickens is less clear. The caecum is involved in water absorption and nitrogen metabolism [1]; however, the caecum can be ligated or completely removed by surgery without any gross effect on health [2]. There are additional differences between characteristics of the small intestine and caecum in chickens. Digestion in the small intestine is continuous with peristalsis [3], while digestion in the caecum is discontinuous, as the caecum is filled and voided approximately twice a day [4]. While there is a concentration of residual oxygen in the small intestine, conditions in the caecum are strictly anaerobic. The small intestine and caecum also differ in the density of colonising microbial populations. The small intestine is sparsely populated with a total bacteria count of approximately 10^5^ per g of digesta, and the microbiota of the adult chicken small intestine consists of *Lactobacilli*, though in young animals, *Romboutsia* or *Turicibacter* may dominate in some individuals [5]. Microbial density and complexity considerably increase in the caecum, reaching total bacterial counts of approximately 10^10^ per g. Due to its high complexity, it is impossible to specify a few species dominating in the caecum, but at the family level, *Lachnospiraceae*, *Ruminococcaceae,* and *Bacteroidaceae* represent the most frequent colonisers of the chicken caecum [6]. Both specialised functions in nutrient absorption and interactions with gut microbiota, therefore, should affect gene expression in these two compartments of the digestive tract.

Microbiota in the densely populated caecum provide its host with lactate, acetate, propionate, or butyrate via digestion and fermentation of complex polysaccharides, which cannot be degraded by host digestive enzymes. Although this part of microbiota activity is considered beneficial for its host, gut microbiota metabolism is also a source of potentially harmful substances, which need to be further metabolised by the host, e.g., methanethiol, the product of microbial methionine catabolism [7]. Moreover, intestinal microbiota, as any other prokaryotic organism, represents a source of microbe-associated molecular patterns (MAMPs), which are sensed by the host through so-called pattern recognition receptors and may stimulate the immune system eventually leading to undesirable inflammation. Finally, conditions in the caecum are characterised by low oxygen levels, which place unusual metabolic demands on the colonic epithelial cells that act at a lower partial pressure of oxygen than other tissues [8]. This results in additional adaptation of the colonic epithelium to “physiologic hypoxia” [9]. Many details concerning the caecum physiology, its response to commensal bacteria, their metabolic products, and all of this operating under low oxygen availability, are not yet fully understood. Studies concerning the diverse aspects of intestinal physiology are therefore needed to serve as a background for future functional studies.

To differentiate between specific functions of the ileum and the caecum, in this study, we identified and compared proteins expressed in the chicken ileum and caecum. Using an unbiased LC-MS/MS protocol, we found that proteins specific to the ileum were those required for nutrient digestion and absorption. On the other hand, proteins characteristic of the caecum were involved in detoxification, hydrogen sulphide (H_2_S) metabolism, cell motility associated with wound healing, and suppression of oxidative stress and inflammatory response.

## 2. Materials and Methods

### 2.1. Origin of Chicken Samples

Newly hatched male ISA Brown chicks were obtained from a local commercial hatchery on the day of hatching. Chicks were reared in perforated plastic boxes of 2 m^2^ with free access to water and standard starter feed, i.e., not sterilised. No specific feed additives or therapeutics were used. The temperature was set to 30 °C during the first week of life.

Altogether, 18 ileal and 57 caecal samples were processed and analysed. All the caecum samples were taken from the middle part of the organ. The ileum was sampled approximately 10 mm proximal to the ileocaecal junction. Following necropsy, the samples were stored in RNA later at −70 °C before parallel RNA and protein purification.

### 2.2. Protein and RNA Purification from Chicken Caecal and Ileal Tissue

Samples of chicken caecal and ileal tissues (50–100 mg) were homogenised in TRI Reagent (MRC) and RNA, and proteins were recovered from upper water and lower phenolic phase, as previously described [10]. mRNA was immediately reverse transcribed into cDNA using M-MLV reverse transcriptase (Invitrogen) and oligo (dT) primers.

### 2.3. Protein Mass Spectrometry

Acetone precipitated protein pellets were dissolved in 300 μL of 8 M urea and processed according to the FASP protocol [11] as described elsewhere [12].

LC–MS/MS analysis of resulting tryptic peptides was performed using an UltiMate 3000 RSLC liquid chromatograph (Dionex) connected to an LTQ-Orbitrap Velos Pro mass spectrometer (Thermo Scientific). Raw LC–MS/MS data were analysed using Proteome Discoverer v1.4. MS/MS, and spectra identifications were performed by the SEQUEST algorithm using the chicken protein sequence database. Only peptides with a false discovery rate of ≤ 5% were considered.

### 2.4. Quantitative Reverse-Transcribed PCR (qRT-PCR)

cDNA was diluted 10 × with sterile water prior to real-time PCR. qRT-PCR was performed in 3 μL volumes in 384-well microplates using QuantiTect SYBR Green PCR Master Mix (QIAGEN) and a NanoDrop pipetting station (Inovadyne) for PCR mix dispensing, as described previously [12]. The Ct values of the genes of interest were normalised (ΔCt) to a geomean Ct value of 3 reference genes, TBP1, HMBS, and ADA, and the relative expression of each gene of interest was calculated as 2^−ΔCt^. The house-keeping reference genes were chosen out of 9 candidates using NormFinder software [13]. All the primers are listed in Supplementary File S1.

### 2.5. Statistics

To identify differentially expressed proteins, peptide spectrum matches (PSMs) counts were used to calculate protein abundance as a percentage in each sample. Only proteins of abundance higher than 0.05% were included in the subsequent analysis. In order to be robust in analysis and to identify the most prominent specific functions of each organ, protein expression was considered as organ specific for the ileum or caecum when showing a ≥3-differential fold ratio (DFR), and a Mann–Whitney test *p*-value was lower than 0.05.

Mann–Whitney test was used for the comparison of the transcriptional values in the caecum and ileum determined by qRT PCR. Genes were considered as differentially expressed in these tissues when *p* < 0.05.

### 2.6. Ethics Approval and Consent to Participate

The handling of animals was performed in accordance with current Czech legislation (Animal Protection and Welfare Act No. 246/1992 Coll. of the Government of the Czech Republic). The specific experiments were approved by the Ethics Committee of the Veterinary Research Institute, followed by the Committee for Animal Welfare of the Ministry of Agriculture of the Czech Republic (permit number MZe1922).

## 3. Results

### 3.1. Gross Comparison of Protein Expression in the Ileum and Caecum

**Figure 1:**
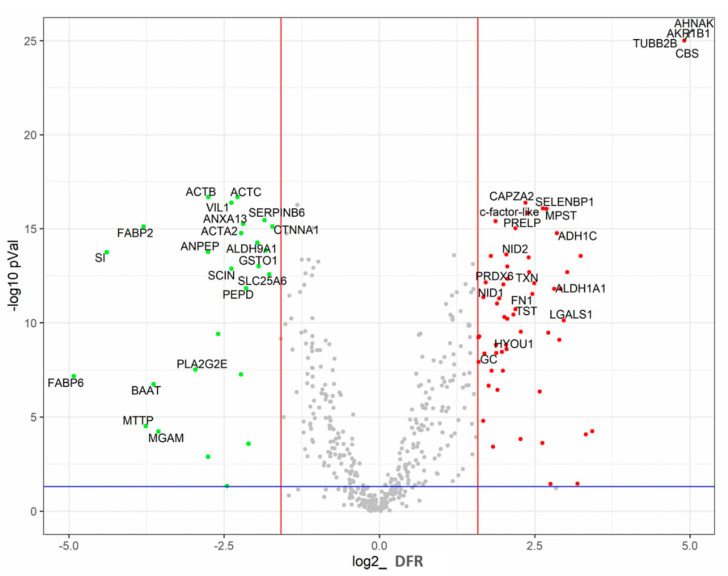
Volcano plot showing protein expressed in the caecum and ileum. Differential expressions presented as log2 of the ratio (X axis) are plotted against *p* values of each comparison. Coloured dots represent proteins fulfilling a threefold difference in abundance in the caecum or ileum and *p*-value < 0.05: green dots—proteins more abundant in the ileum; red dots—proteins more abundant in the caecum. Overall, 20 differentially expressed and the most abundant proteins in the ileum or caecum are identified. For the whole dataset, see Supplementary File S2. Position of FABP6 as ileum-specific protein and AHNAK, AKR1B1, TUBB2B, and CBS as caecum-specific proteins along the X axis does not represent their differential fold ratio as these proteins were highly specific for the ileum or caecum, respectively (see text below).

Altogether, 11,361 chicken proteins were detected. However, when only the proteins representing at least 0.05% of total protein in at least one of the two tissues were considered, the number of compared proteins decreased to 484 (Figure 1). Of these, 91 proteins were differentially expressed; 25 were enriched in the ileum and 66 in the caecum. Nine caecum-specific proteins were expressed exclusively in the caecum, i.e., were not detected in the ileum at all, while there was not one protein exclusively specific for the ileum.

### 3.2. Ileum-Specific Proteins

Four proteins specific to the ileum were of notably higher abundance compared with the remaining ileal proteins. These included three actins—ACTA2, ACTB, and ACTC—and fatty acid-binding protein 6 (FABP6). Each of these proteins formed more than 4% of total ileal proteins (Table 1). Functionally, the 20 most abundant ileum-specific proteins could be grouped into proteins forming cytoskeleton or involved in digestion, and lipid and bile acid metabolism (Table 2). The expression of six ileum-specific proteins was verified at the mRNA level by qRT-PCR. Four of them (FABP6, VIL1, SI, and BAAT) were confirmed to be differentially expressed in the ileum and caecum, also at mRNA level, while differential expression at the transcriptional level was not confirmed for ACTB and CTNNA1 (Table 1).

### 3.3. Caecum-Specific Proteins

In total, 66 proteins were identified as caecum specific. Expression of the caecum-specific proteins represented 11.70% of total caecal proteins. Galectin 1 (GAL-1) was the protein with the highest abundance in the caecum, followed by thioredoxin (TXN) and c-factor-like protein. The expression of 11 caecum-specific proteins was verified at the mRNA level by qRT-PCR, and all of them were confirmed to be differentially expressed in the caecum and ileum (Table 3).

### 3.4. Functional Analysis of Caecum-Specific Proteins

**Figure 2:**
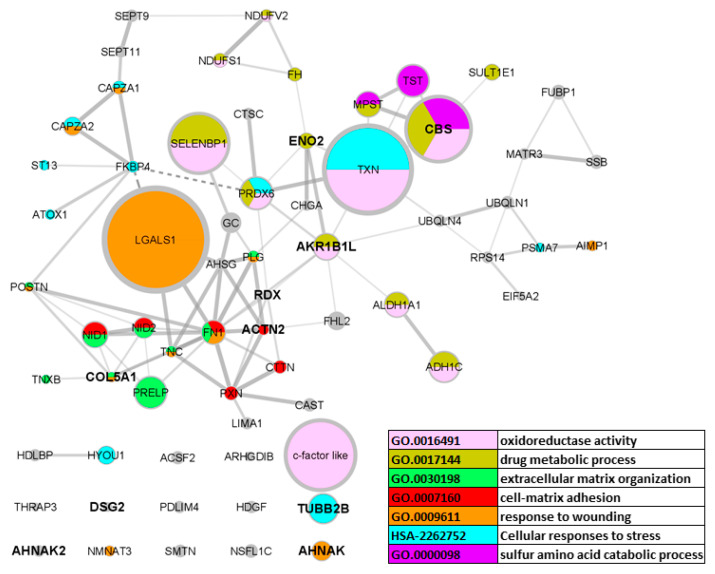
Caecum-specific proteins network generated by STRING. Size of the nodes is proportional to the caecum protein expression. Caecum exclusive proteins, i.e., proteins not expressed in the ileum at all, are highlighted in bold.

To identify enriched sets of proteins, the caecum-specific gene set was analysed by STRING [14]. A total of 12 proteins (AKR1B1L, ALDH1A1, PRDX6, MPST, CBS, ADH1C, SELENBP1, SULT1E1, NDUFV2, FH, NDUFS1, ENO2) were identified as related to drug metabolic processes and metabolism of exogenous substances. Of these, AKR1B1L, ALDH1A1, PRDX6, MPST, CBS, ADH1C, and SELENBP1 represent different oxidoreductases (Figure 2), which indicates that the majority of exogenous substances are degraded by oxidation/reduction processes. These proteins represented 22.48% of caecum-specific proteins (Table 4).

Overall, 12 proteins (GAL-1, FN1, AHNAK, CAPZA2, FHL2, CAPZA1, PLG, TNC, NMNAT3, AIMP1, COL5A1, POSTN) were identified as involved in wound healing. These proteins represented 17.39% of caecum-specific proteins.

Further, 14 proteins (FN1, NID2, NID1, PRELP, TNC, PLG, COL5A1, POSTN, AIMP1, CAST, TNXB, PXN, CTTN, ACTN2) belonged to an extracellular matrix organisation and cell–matrix adhesion proteins. These proteins represented 13.52% of caecum-specific proteins. Five of those proteins (PRELP, FN1, COL5A1, POSTN, and TNXB) bind sulphated glycosaminoglycans, a family of complex polysaccharides found in the extracellular matrix and on cell surfaces.

Another enriched group of proteins included those involved in the cellular response to stress (TXN, TUBB2B, PRDX6, CAPZA2, CAPZA1, ATOX1, ST13, TNXB). Proteins in this category represented 11.59% of all caecum-specific proteins.

Finally, there was a group of three proteins—CBS, MPST, and TST—identified as related to the catabolism of sulphur-containing amino acids. Together with methanethiol oxidase SELENBP1 [7], these proteins are related to H_2_S metabolism. Since these proteins ranked 4, 5, 9, and 13 among the most abundant caecum-specific proteins, representing 8.71% of caecum-specific proteins, H_2_S release and detoxification is an additional important function of the caecal tissue (Table 4, Figure 2).

### 3.5. Expression of Key Regulatory Proteins of Oxidative Stress

**Figure 3:**
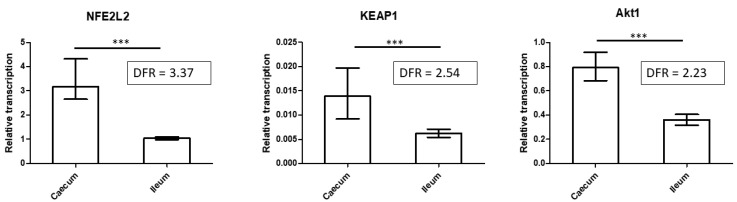
mRNA expression of NFE2L2, KEAP1, and AKT1 in the caecum and ileum. A higher level of transcription of NFE2L2, KEAP1, and AKT1, oxidative stress transcriptional regulators, was recorded in the caecum than in the ileum (Mann–Whitney, *** indicate *p* < 0.0005). DFR = differential fold ratio.

As multiple caecum-specific proteins were related to redox sensing and response, expression of the main oxidative stress transcriptional factor NFE2L2, its directly regulated cytoplasmic repressor Keap1, and functionally related AKT1 were determined by qRT PCR. As expected, all three genes were significantly more transcribed in the caecum than in the ileum (Figure 3).

## 4. Discussion

To better understand differences in the caecum and ileum functions in chickens, protein expression in these two parts of the intestinal tract was determined by mass spectrometry. There has been a similar study which utilised RNA sequencing to compare transcriptome in the jejunum and caecum [15]. Despite methodological differences, SI, MGAM, and FABP2 were identified as proteins specific for the small intestine, and CBS, c-factor-like protein, and SELENBP1 were similarly identified as genes specifically expressed in the caecum in both studies.

Over 73% of ileum-specific proteins represented either structural or enzymatic components of the apical part of the intestinal epithelium. Three actins (ACTA2, ACTB, and ACTC), villin (VIL1), myosin-1a (MYO1A), and adseverin (SCIN) are responsible for the morphology of enterocyte microvilli [16]. Four digestion enzymes (SI, MGAM, PEPD, ANPEP) are also typical for enterocyte brush border [17,18]. SI and MGAM work concurrently to hydrolyse the mixture of oligosaccharides resulting from starch digestion to glucose and other monosaccharides [19], and ANPEP and PEPD ensure peptide digestion. Another group of ileum-specific proteins was involved in lipid and bile acid metabolism. The role of ileum in bile acid metabolism is known and the specificity of BAAT or FABP6 for ileum has been reported [20]. The chicken ileum is therefore the site of efficient nutrient digestion and absorption.

The function of chicken caecal tissue was more complex. Oxidative reduction events, detoxification, H_2_S metabolism, and wound healing were the functions, which differentiated the caecum from the ileum. Due to the difference in microbiota density and composition in the ileum and caecum and the fact that the expression of some of these proteins is influenced by gut microbiota [10,12], we propose that expression of these systems is at least partially influenced by MAMP signalling pathways and metabolism of microbiota metabolic products.

Aldehyde dehydrogenase 1 (ALDH1A1) is a detoxifying enzyme which catalyses the oxidation of exogenous and endogenous aldehyde substrates to their corresponding carboxylic acids and is considered a marker of stem cells as well as cancer cells in different tissues including the colon [21,22]. Alcohol dehydrogenase, aldo–keto reductase (ADH1C, AKR1B1L), and likely also c-factor-like protein (based on similarity) [23,24,25,26] represent enzymes which are closely related to ALDH1A1. TXN and PRDX6 suppress oxidative stress caused by reactive oxygen species by preventing protein oxidation by cysteine thiol–disulphide exchange [27,28] and CBS-producing L-cystathionine protects against stress-induced cell death [29,30].

Beta-galactoside-binding lectin (galectin-1; GAL-1) represented the most abundant caecum-specific protein, similar to a report in humans [31]. Galectin-1 is involved in interactions between cells and the extracellular matrix [32,33] but exhibits also strong immunomodulatory tolerogenic potential [34,35]. This may correspond with a much higher exposure of the caecal epithelium to luminal microbiota. Higher activity in caecal wound healing is documented by specific expression of nidogen-1 and -2 which connect laminin and collagen IV networks in basement membranes [36,37,38] or fibronectin involved in cell adhesion, motility, and migration associated with wound healing [39]. An additional set of caecum-specific proteins associated with epithelium integrity was involved in cell migration and coordinated epithelial sheet motility. These included AHNAK, CAPZA2, CAPZA1, RDX, CTTN and FHL2 [40,41,42,43,44,45]. Furthermore, TNX, ENO2, and CHGA are exclusively expressed by neuronal and neuroendocrine cells [46,47], and their higher abundance in the caecum reflects differences in innervation as well as in the neuroendocrine cell distribution between small and large intestine observed earlier [48].

The role of H_2_S in the colon/caecum is not fully understood. Although negative effects of high levels of H_2_S produced by enteric bacteria were reported [49,50], there are numerous studies demonstrating protective effects of H_2_S against chemical and oxidative stress in induced GI tract injury, as well as its role in promoting resolution of inflammation and repair of tissue damage [51,52,53]. H_2_S also regulates host–microbiota cohabitation by promoting mucus secretion and microbiota biofilm formation [54]. Additionally, cytoprotective transcriptional regulators NFE2L2 [55] and AKT1 were transcribed at a higher level in the caecum than in the ileum. Since NFE2L2 is activated in conditions of oxidative stress [56], and the AKT1 pathway plays a major role in cell survival [57], this further confirms the higher need for stress-suppressing mechanisms in the caecum than in the ileum.

## 5. Conclusions

At an early age, the chicken caecum, compared with the ileum, has a higher capacity to sense and limit the consequences of oxidative stress caused at least partially by exposure to a complex mixture of MAMPs and gut microbiota metabolism products. This likely causes micro-disruptions of the intestinal barrier, which is counter-balanced by increased cell motility associated with epithelium renewal. This is coupled with the activation of mechanisms suppressing the inflammatory response. Collectively, the obtained results show that the caecum is responsible for more than mere water absorption and nitrogen metabolism.

## Figures and Tables

**Table 1:** Top 20 ileum-specific proteins ranked by abundance.

Protein Annotation	Protein	*p*-Value	Abundance in Ileum [%]	DFR *	qRT-PCR Fold
ENSGALP00000010239	ACTA2	1.73 × 10^−15^	4.94	4.67	-^#^
ENSGALP00000039176	ACTB	2.09 × 10^−17^	4.82	6.75	NS
ENSGALP00000018042	ACTC	2.09 × 10^−17^	4.64	4.87	-
ENSGALP00000002196	FABP6	6.95 × 10^−8^	4.12	311.34	2.43
ENSGALP00000037398	VIL1	4.18 × 10^−17^	0.60	5.21	6.43
ENSGALP00000019528	FABP2	7.73 × 10^−16^	0.50	13.89	-
ENSGALP00000015467	SI	1.78 × 10^−14^	0.40	20.94	313.41
ENSGALP00000003847	CTNNA1	7.72 × 10^−16^	0.27	3.30	NS
ENSGALP00000037173	SCIN	1.33 × 10^−13^	0.26	5.21	-
ENSGALP00000005520	ALDH9A1	5.55 × 10^−15^	0.2	3.90	-
ENSGALP00000020953	SERPINB6	3.45 × 10^−16^	0.17	3.61	-
ENSGALP00000000256	MGAM	5.94 × 10^−5^	0.16	11.77	-
ENSGALP00000007819	PEPD	1.48 × 10^−12^	0.13	4.42	-
ENSGALP00000032098	BAAT	1.85 × 10^−7^	0.12	12.42	7.00
ENSGALP00000035815	SLC25A6	2.67 × 10^−13^	0.12	3.42	-
ENSGALP00000008315	ANPEP	1.68 × 10^−14^	0.10	6.76	-
ENSGALP00000013682	GSTO1	9.86 × 10^−14^	0.10	3.85	-
ENSGALP00000022884	PLA2G2E	3.05 × 10^−8^	0.09	7.81	-
ENSGALP00000026341	ANXA13	5.55 × 10^−16^	0.09	4.58	-
ENSGALP00000036834	MTTP	3.05 × 10^−5^	0.09	13.57	-

* DFR—differential fold ratio of protein abundance in the ileum compared to caecum. # “-“ = not tested, NS = not significant.

**Table 2:** Ileum-specific proteins grouped according to function.

Group Description	No. of Proteins	% Expression out of Ileum-Specific Proteins	Proteins
Actin and actin-binding	7	70.13	ACTA2; ACTB; ACTC; VIL1; CTNNA1; SCIN; MYO1A
Bile metabolism and transport	2	19.04	FABP6; BAAT
Digestion enzymes	4	3.53	SI; MGAM; PEPD; ANPEP
Lipid metabolism and transport	4	3.51	FABP2; PLA2G2E; MTTP; PLIN4
Other	8	3.80	SERPINB6; ANXA13; GSTO1; SLC25A6; RPS8; RPS8; GDA; SORD

**Table 3:** The top 20 proteins specific for the caecum ranked according to abundance.

Protein Annotation	Protein	*p*-Value	Abundance in Caecum [%]	DFR *	qRT-PCR Fold	Influenced by Microbiota Colonisation ^#^
ENSGALP00000020275	GAL-1	7.49 × 10^−11^	0.63	7.83	2.40	
ENSGALP00000025280	TXN	7.89 × 10^−13^	0.59	5.62	6.39	UP
ENSGALP00000001060	c-factor-like	8.37 × 10^−17^	0.48	6.20	-	
ENSGALP00000026062	CBS	NA	0.43	500.00	3488.35	UP
ENSGALP00000001274	SELENBP1	8.72 × 10^−17^	0.36	6.44	14.72	
ENSGALP00000019979	ADH1C	1.73 × 10^−15^	0.21	7.24	31.09	DOWN
ENSGALP00000036423	TUBB2B	NA	0.20	500.00	-	
ENSGALP00000004816	PRDX6	7.23 × 10^−13^	0.20	3.28	13.14	
ENSGALP00000029947	TST	3.65 × 10^−11^	0.19	4.47	4.90	UP
ENSGALP00000017869	NID1	5.04 × 10^−12^	0.18	3.81	-	
ENSGALP00000005480	AKR1B1L	NA	0.18	500.00	-	DOWN
ENSGALP00000005607	PRELP	9.41 × 10^−16^	0.18	4.56	-	
ENSGALP00000024396	ALDH1A1	1.58 × 10^−12^	0.17	7.02	16.55	DOWN
ENSGALP00000005654	FN1	2.98 × 10^−12^	0.16	5.50	2.37	
ENSGALP00000020214	NID2	2.37 × 10^−14^	0.14	4.12	-	
ENSGALP00000020373	MPST	8.72 × 10^−17^	0.14	6.46	7.43	
ENSGALP00000023289	AHNAK	NA	0.12	500.00	2.19	
ENSGALP00000039757	HYOU1	2.52 × 10^−9^	0.12	4.13	-	
ENSGALP00000015277	CAPZA2	4.18 × 10^−17^	0.11	5.11	-	
ENSGALP00000018939	GC	4.16 × 10^−9^	0.11	3.68	-	DOWN

* DFR—differential fold ratio of protein abundance in the caecum compared to ileum. Value 500 was given to proteins expressed exclusively in the caecum and not in the ileum. #—compared with data in reference [10].

**Table 4:** Caecum-specific proteins grouped according to function.

Group Description	No. of Proteins	% Expression out of Caecum-Specific Proteins	Proteins
Drug metabolic processes and metabolism of exogenous substances	12	22.48	AKR1B1L, ALDH1A1, PRDX6, MPST, CBS, ADH1C, SELENBP1, SULT1E1, NDUFV2, FH, NDUFS1, ENO2
Wound healing	12	17.39	GAL-1, FN1, AHNAK, CAPZA2, FHL2, CAPZA1, PLG, TNC, NMNAT3, AIMP1, COL5A1, POSTN
Extracellular matrix organisation and cell–matrix adhesion proteins	14	13.52	FN1, NID2, NID1, PRELP, TNC, PLG, COL5A1, POSTN, AIMP1, CAST, TNXB, PXN, CTTN, ACTN2
Cellular response to stress	8	11.59	TXN, TUBB2B, PRDX6, CAPZA2, CAPZA1, ATOX1, ST13, TNXB
H_2_S metabolism	4	8.71	CBS, MPST, TST, SELENBP1

## Data Availability

Data generated or analysed during this study are included in this published article and its Supplementary Information Files.

## Supplementary Materials

The following are available online at https://www.mdpi.com/article/10.3390/ani11113155/s1. Supplementary File S1: Primers used in the study—The list of all primers used and referred to in the study, Supplementary File S2: Protein expression data—Table of protein abundance expressed as a percentage in each sample. Only proteins of abundance higher than 0.05% are shown.

## Abbreviations

**ACTA2**: Actin alpha 2; **ACTB**: Actin beta; **ACTC**: Actin alpha cardiac; **ACTN2**: Actinin alpha 2, **ADH1C**: Alcohol dehydrogenase 1C; **AHNAK**: AHNAK nucleoprotein, Desmoyokin; **AIMP1**: Aminoacyl TRNA synthetase complex interacting multifunctional protein 1; **ANPEP**: Membrane alanyl aminopeptidase; **AKR1B1L**: Aldo-keto reductase family 1, member B1-like; **AKT1**: AKT serine/threonine kinase 1; **ALDH1A1**: Aldehyde dehydrogenase 1 family member A1; **ATOX1**: Antioxidant 1 copper chaperone, **BAAT**: Bile acid-CoA:amino acid N-acyltransferase; **CAPZA1**: Capping actin protein of muscle Z-line subunit alpha 1; **CAPZA2**: Capping actin protein of muscle Z-line subunit alpha 2; **CAST**: Calpastatin; **CBS**: Cystathionine beta-synthase; **COL5A1**: Collagen type V alpha 1 chain; **CTNNA1**: Catenin alpha 1; **CTTN**: Cortactin; **ENO2**: Enolase 2; **FABP6**: Fatty acid binding protein 6; **FH**: Fumarate hydratase; **FHL2**: Four and a half LIM domains 2; **FN1**: Fibronectin 1; **GAL-1**: Galectin 1; **KEAP1**: Kelch like ECH associated protein 1; **MGAM**: Maltase-glucoamylase; **MPST**: Mercaptopyruvate sulphurtransferase; **NDUFS1**: NADH:ubiquinone oxidoreductase core subunit S1; **NDUFV2**: NADH:ubiquinone oxidoreductase core subunit V2; **NFE2L2**: Nuclear factor, erythroid 2 like 2; **NID1**: Nidogen 1; **NID2**: Nidogen 2; **NMNAT3**: Nicotinamide nucleotide adenylyltransferase 3; **PEPD**: Peptidase D; **PLG**: Plasminogen; **POSTN**: Periostin; **PRDX6**: Peroxiredoxin 6; **PRELP**: Proline and arginine rich end leucine rich repeat protein; **PXN**: Paxillin; **SELENBP1**: Selenium binding protein 1; **SI**: Sucrase-isomaltase; **ST13**: ST13 Hsp70 interacting protein; **SULT1E1**: Sulphotransferase family 1E member 1; **TNC**: Tenascin C; **TNXB**: Tenascin XB; **TST**: Thiosulphate sulphurtransferase; **TUBB2B**: Tubulin beta 2B class IIb; **TXN**: Thioredoxin; **VIL1**: Villin 1
